# Impact of Primary Breast Surgery on Overall Survival of Patients With De Novo Metastatic Breast Cancer: A Systematic Review and Meta-Analysis

**DOI:** 10.1093/oncolo/oyad266

**Published:** 2023-09-12

**Authors:** Guillermo Villacampa, Andri Papakonstantinou, Irma Fredriksson, Alexios Matikas

**Affiliations:** SOLTI Breast Cancer Research Group, Spain; The Institute of Cancer Research, London, UK; Oncology Data Science, Vall d’Hebron Institute of Oncology (VHIO), Barcelona, Spain; Oncology/Pathology Department, Karolinska Institute, Stockholm, Sweden; Breast Center, Karolinska University Hospital and Karolinska Comprehensive Cancer Center, Stockholm, Sweden; Breast Center, Karolinska University Hospital and Karolinska Comprehensive Cancer Center, Stockholm, Sweden; Department of Molecular Medicine and Surgery, Karolinska Institute, Stockholm, Sweden; Oncology/Pathology Department, Karolinska Institute, Stockholm, Sweden; Breast Center, Karolinska University Hospital and Karolinska Comprehensive Cancer Center, Stockholm, Sweden

**Keywords:** breast cancer, breast surgery, meta-analysis, metastatic, overall survival

## Abstract

**Background:**

Breast surgery in cases of de novo metastatic breast cancer (MBC) is associated with improved outcomes in retrospective studies, although the results of randomized controlled trials (RCTs) are conflicting. We aimed to investigate whether surgery in this context prolongs patient survival.

**Methods:**

We performed a systematic review of the literature to identify RCTs comparing surgery of primary breast cancer to no surgery in patients with de novo MBC. Cochrane Library, Embase, Medline (OVID), and Web of Science were searched with latest update in July 2023, while conference proceedings were manually searched. Data concerning patient and tumor characteristics, as well as outcomes, were extracted. A meta-analysis with random effects models was performed considering heterogeneity between trials.

**Results:**

Overall, 3255 entries were identified and 5 RCTs fulfilled all inclusion criteria, which had enrolled 1381 patients. The overall estimation in the intention-to-treat population showed no benefit for patients who had surgical excision of the primary breast tumor (HR = 0.93; 95% CI, 0.76-1.14). No subgroups in terms of receptor status or patterns of metastasis seemed to benefit from surgery, except for younger/premenopausal patients (HR = 0.74, 95% CI, 0.58-0.94). Breast surgery was associated with improved local progression-free survival (HR = 0.37, 95% CI, 0.19-0.74).

**Conclusion:**

Surgery of the primary tumor in patients with de novo MBC does not prolong survival, except possibly in younger/premenopausal patients. Breast surgery should be offered within the context of well-designed clinical trials examining the issue.

## Teaser

Breast surgery in cases of de novo metastatic breast cancer is associated with improved outcomes in retrospective studies, but results of randomized controlled trials are conflicting. This systematic review and meta-analysis investigated whether surgery in this context prolongs patient survival.

**Implications for Practice**

Due to methodological weaknesses and conflicting results from prospective trials, as well as support from retrospective studies, removal of primary tumor in patients with metastatic breast cancer persists to this day. By summarizing all available data from the 5 randomized trials examining the issue, we conclude that surgical excision of the primary tumor in case of de novo metastatic breast cancer is not associated with improved patient survival. As such, besides the need to palliate local symptoms, surgery should not be routinely offered to patients with metastatic disease.

## Introduction

Breast cancer presents with disseminated disease at the time of, or within 3 months from, diagnosis in approximately 3%-10% of patients.^1-3^ Patients with de novo metastatic breast cancer (MBC) have better prognosis compared to those with distant recurrence following primary treatment for early disease,^4,5^ presumably due to the selection and expansion of resistant clones in the latter case. Considering the prolonged natural history of de novo MBC, strategies for locoregional control are theoretically appealing to prevent continuous seeding and further metastatic spread promoted by mesenchymal stem cells in primary breast tumors.^6^ Indeed, retrospective studies have shown a potential survival benefit with breast surgery.^7^ However, selection bias is inherent in such studies, since younger, more fit patients with indolent disease course might be more likely to be offered breast surgery.^8^

To definitively answer the question whether to perform surgical excision of the primary tumor in case of de novo MBC, 5 randomized controlled trials (RCTs) have been conducted.^9-13^ Differences in study design including timing of surgery, methodological issues, moderate sample sizes, and conflicting results weaken the available evidence. In addition, individual studies were underpowered to analyze the benefit of surgery in different clinically relevant subgroups. This ambiguity is clearly reflected in contemporary treatment guidelines. Although 4 of 5 RCTs have not demonstrated any survival benefit, surgery is recommended for selected patients according to some,^14^ but not all resources.^15^ This has in turn led to a continuous use of aggressive locoregional control in clinical practice. For example, a large retrospective study conducted during the era of modern systemic therapies reported that one out of 4 patients with de novo MBC had breast surgery within 12 months from diagnosis, and that these patients had improved overall survival (OS) in propensity score matching analysis.^16^

Considering the above, the question regarding the benefit of surgical removal of the primary tumor in de novo MBC remains largely unanswered, as benefit from surgery limited to patient subgroups has hitherto not been possible to exclude in individual trials. As retrospective studies suffer from selection bias due to unknown confounders and individual prospective RCTs might be inadequately powered to detect small differences in favor of surgery, we pooled the available data from all reported RCTs with the aim to provide a definitive answer to this question.

## Methods

### Search Strategy and Study Selection

We performed a systematic review of the literature to identify RCTs comparing surgical removal of the primary breast tumor versus no surgery in patients with de novo MBC. The study selection and meta-analysis were conducted and reported according to the Preferred Reporting Items for Systematic Reviews and Meta-Analyses (PRISMA) guidelines (Supplementary Material).^17^ The present study was registered in the international prospective register of systematic reviews PROSPERO, with registration number CRD42023430327.

To be included in the systematic review, potentially eligible studies had to meet the following criteria: (1) randomized controlled trial, (2) patients with de novo MBC, (3) at least one treatment group with surgical removal of the primary breast tumor, (4) information of any efficacy endpoint was available, and (5) publication in English. Retrospective studies, prospective single-arm studies, or meta-analyses were not included, and neither were ongoing studies with unpublished results at the time of the literature search were.

A search of the following databases was performed by 2 librarians at the Karolinska Institute Library in December 2022 and updated in July 2023: Cochrane Library, Embase, Medline (OVID), and Web of Science. The search strategy was developed in Medline (Ovid). For each search concept, Medical Subject Headings (MeSH-terms) and free text terms were identified. The search was then translated, in part using Polyglot Search Translator,^18^ into the other databases. Databases were searched from inception. The strategies were peer reviewed by another librarian prior to execution. De-duplication was done using the method described by Bramer et al.^19^ One final, extra step was added to compare DOIs. The full search strategies for all databases are available as Supplementary Material. A review of conference proceedings from the European Society of Medical Oncology (ESMO) congress, the American Society of Oncology (ASCO) annual meeting, and San Antonio Breast Cancer Symposium was also conducted to identify relevant unpublished studies and to include the most up-to-date results. Selection and examination of potentially eligible full articles were performed by A.M. A risk of bias assessment was performed for the primary outcome (OS) using the revised Cochrane Risk of bias assessment of randomized trials tool (RoB2).^20^

### Data Extraction

The following variables were extracted to a predefined form, if available: name of the study, Clinicaltrials.gov identifier, name of first author, journal and year of publication, total sample size, sample size per treatment arm, time of randomization (at inclusion or following systemic therapy), median follow-up, type of assessment to document disease progression, hazard ratio (HR) with associated 95% confidence intervals (CI) for local progression-free survival (LPFS), progression-free survival (PFS), and for OS, number of patients and corresponding HR per disease site (bone vs. visceral disease), number of patients and HR per hormone receptor and HER2 status (positive vs. negative), number of patients and HR per age group, number of patients and HR per group based on number of metastatic sites. Finally, the number of patients allocated to systemic therapy only but operated and number of patients allocated to surgery but also not operated were collected. Variables were extracted by 2 authors independently (A.P. and A.M.), and discrepancies were discussed until consensus was reached.

### Outcomes

The primary endpoint was OS, defined as the time from randomization to death from any cause. Subgroup analysis was performed to identify the effect of breast surgery on OS in specific subgroups of interest defined by age (younger vs. older age), tumor characteristics (hormone receptor and HER2 status), and pattern of metastasis (visceral vs. bone-only disease, oligometastatic vs. non-oligometastatic disease). Age groups were not consistently defined across included studies, with some having an age cutoff^10,12^ and others using menopausal status,^9,12,13^ while ABCSG-28 reported no relevant subgroup analysis.^11^ Within the scope of the present analysis, premenopausal patients and patients under the specific for each study age cutoff were pooled together, as well as postmenopausal ones with those over the study-specific age cutoff. The secondary endpoint was LPFS, defined as time from randomization to first locoregional progression or recurrence or death from any cause, whichever occurred first. Finally, the third endpoint of PFS was a composite of the overall PFS and distant PFS endpoints reported in 3 of the 5 RCTs.^9,11,13^

### Statistical Analysis

Hazard ratios (HRs) with 95% confidence intervals (95% CI) were calculated to summarize the trial-level meta-analysis for the OS, LPFS, and PFS endpoints. HR < 1 indicates a risk reduction for survival outcomes (protective effect) with breast surgery. Random effects models using the DerSimonian-Laird method for pooling were used to calculate the overall HR assuming that there is not one true intervention effect but a distribution of true intervention effect. The use of random effects was motivated by the heterogeneity between trials (induction chemotherapy vs. upfront surgery). As a sensitivity analysis pooled random model analysis removing each study, one by one, and repeating the meta-analysis was reported to assess the influence of each study.

Heterogeneity estimation was calculated and reported in all analysis by means of *I*^2^ that estimates the percentage of total variability due to between-studies heterogeneity. Funnel plot analysis and Egger’s test were performed to detect publication bias. All analyses were performed using R statistical software version 4.1.2 (R packages *metafor* and *meta*).

## Results

### Study and Patient Characteristics

The literature search, provided in detail in Supplementary Materials, identified 5581 entries, or 3255 following deduplication. In addition, 328 studies were selected from conference websites. Following screening according to the aforementioned predefined criteria, 5 RCTs examining the impact of primary breast surgery on survival of patients with de novo MBC were included in the analysis, of which 4 have been published in full form, and one was presented as poster at ASCO 2023 annual meeting. For the MF07-01 trial, the latest update after 10 years of follow-up was included to the meta-analysis.^21^ Information on study design and population characteristics is summarized in Table 1. Inclusion criteria were not consistent across the 5 RCTs: 2 studies mandated an initial period of systemic therapy and only enrolled patients without disease progression,^8,13^ 2 offered upfront surgery,^10,11^ while one RCT allowed chemotherapy prior to surgery for patients with initially unresectable tumors, whereas patients with resectable tumors were offered surgery followed by endocrine therapy.^9^ In the studies that enrolled patients after an initial period of systemic therapy, use of chemotherapy and endocrine therapy varied: in the EA2108 study, chemotherapy alone, endocrine therapy alone, and chemotherapy with HER2-blockade were used in about a third of enrolled patients.^12^ In Badwe et al, all patients with initially unresectable disease were treated with chemotherapy.^9^ Finally, in the PRIM-BC study, systemic therapy was described in the protocol and depended on receptor status and presence of life-threatening disease.^13^

**Table 1.** Main characteristics of studies included in the meta-analysis.

First author, year publication	Study name	Identifier	Years of enrollment	Sample size	Median follow-up (months)	ER positive	HER2 positive	Bone-only disease	Timing of surgery	Clear surgical margins	Primary endpoint
Badwe, 2015	Badwe et al	NCT00193778	2005-2013	350	23	59.4%	30.5%	28.5%	At inclusion if resectable, after systemic therapy if unresectable	NR	Overall survival
Soran, 2021 (previously 2018)	MF07-01	NCT00557986	2007-2012	278 (265 in latest analysis)	120	79.2%	29.0%	46.0%	At inclusion	NR	Overall survival
Fitzal 2019	ABCSG-28	NCT01015625	2011-2015	90	37.5	81.1%	22.2%	37.8%	At inclusion	76.2%	Overall survival
Khan, 2022	EA2108	NCT01242800	2011-2015	256	53	59.6% ^*^	32.2%	37.7%	After 16-32 weeks of systemic therapy	91.5%	Overall survival
Shien, 2023	PRIM-BC	UMIN000005586	2011-x	407	60	71.9%	29.7%	28.7%	After 3 months of systemic therapy	87.3%	Overall survival

^*^ER-positive/HER2-negative.

Abbreviations: ER: estrogen receptor; HER2: human epidermal growth factor receptor 2; NR: not reported.

A total of 1381 patients were included in the present meta-analysis, of whom 685 (49.6%) had primary breast surgery and 696 (50.4%) did not. The percentage of patients with bone-only disease was 34.9% (28%-46% across trials), while visceral disease was present 61.3%. The percentage of hormone receptor positive and HER2-positive disease was 67.9% and 25.3%, respectively. Overall, 11.4% of patients allocated to systemic therapy alone received breast surgery, whereas 10.6% of those allocated to surgery were not operated. OS was the primary endpoint in all the studies.

The 4 RCTs that have been published in full form were assessed for bias, with one study showing high risk of bias (Supplementary Fig. S1). Visual inspection of the funnel plot and the Egger’s test revealed no evidence of publication bias (Supplementary Fig. S2).

### Overall Survival

**Figure 1.**
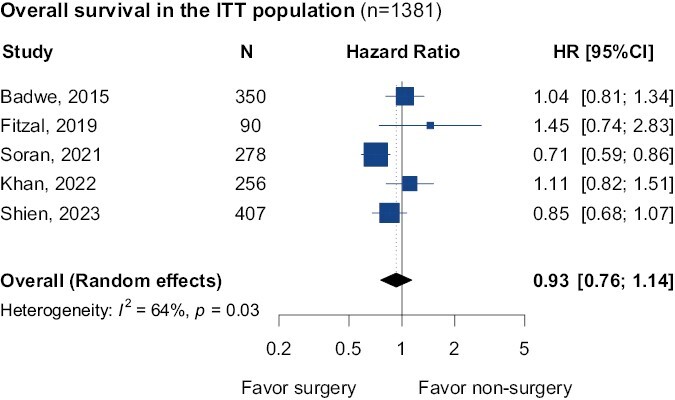
Overall survival pooled results in the intention-to-treat (ITT) population in the comparison of breast surgery vs no breast surgery. Abbreviations: HR: hazard ratio; 95% CI: 95% confidence interval.

For the meta-analysis evaluating OS (*n* = 1381), the overall estimation in the intention-to-treat (ITT) population showed no benefit in patients with surgical removal of the primary breast tumor (HR = 0.93; 95% CI, 0.76-1.14; Fig. 1). The heterogeneity between trial in terms of the *I*^2^ statistic was 64%. Results were consistent in all sensitivity analyses (Supplementary Fig. S3).

**Figure 2.**
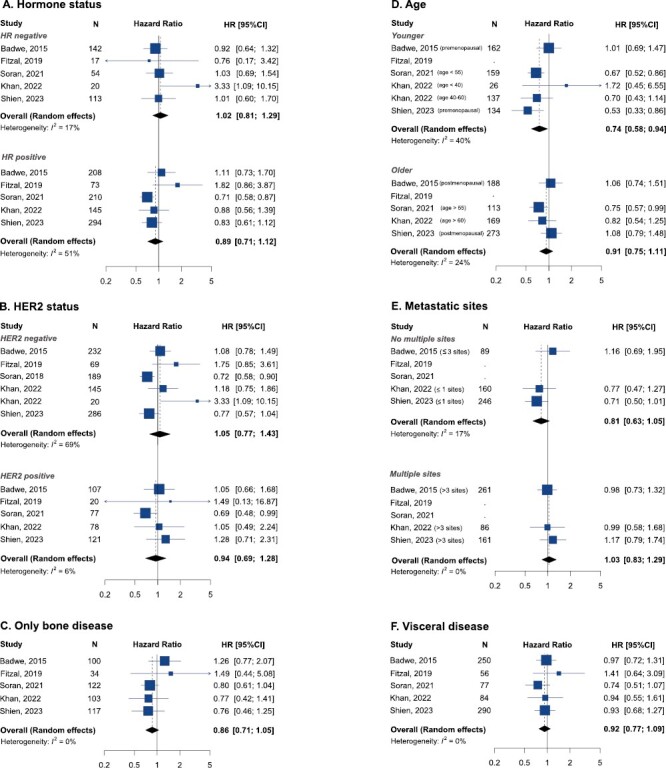
Overall survival pooled result according to subgroup analysis. (**A**) hormone status, (**B**) HER2 status, (**C**) only bone disease, (**D**) age, (**E**) metastatic sites, and (**F**) visceral disease. Abbreviations: HR: hazard ratio; 95% CI: 95% confidence interval.

A subgroup analysis was carried out regarding (1) hormone receptor status, (2) HER2 status, (3) age group, (4) number of metastasis sites, and (5) metastasis location (Fig. 2). No relevant differences were observed by hormone receptor or HER2 status. Patients with hormone receptor negative disease did not benefit from primary surgery (HR = 1.02, 95% CI, 0.81-1.29), nor did those with hormone receptor positive (HR = 0.89, 95% CI, 0.71-1.12), HER2 negative (HR = 1.05, 95% CI, 0.77-1.43) and HER2-positive MBC (HR = 0.94, 95% CI, 0.69-1.28). In the same direction, no differences were observed by metastatic location (bone vs. visceral) or number of metastatic sites (solitary/few vs. multiple; Fig. 2). Benefit from primary surgery could be observed in the subgroup analysis by age. Younger women, in terms of age or premenopausal status, seemed to benefit from removal of the primary tumor (HR = 0.74, 95% CI, 0.58-0.94).

### Local Relapse-Free Survival and Progression-Free Survival

**Figure 3.**
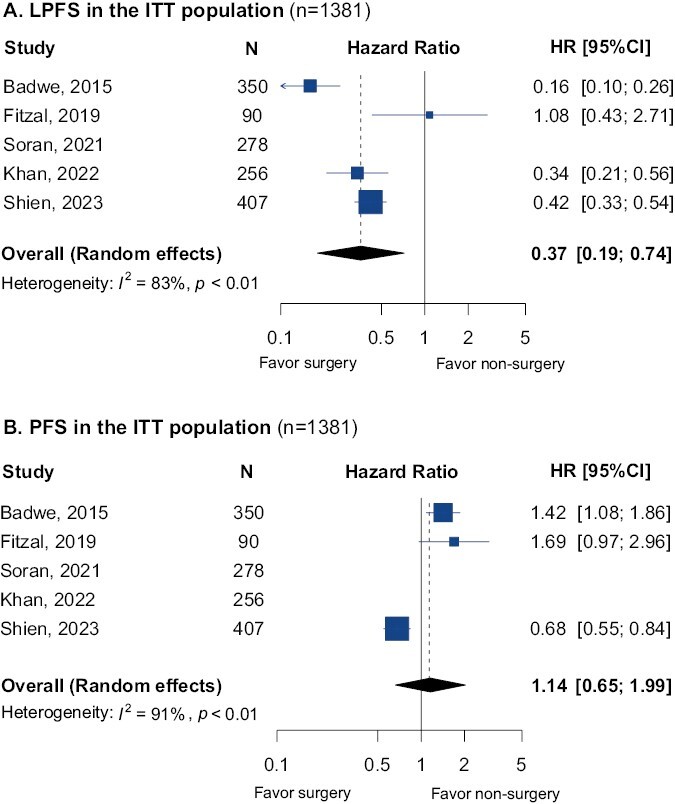
(**A**) Local progression-free survival (LPFS) pooled results in the intention-to-treat (ITT) population in the comparison of breast surgery vs. no breast surgery. (**B**) Progression-free survival (PFS) pooled results in the ITT population in the comparison of breast surgery vs. no breast surgery. Abbreviation: HR: hazard ratio; 95% CI: 95% confidence interval.

The effect of surgical removal of the primary breast tumor on LPFS was reported in 4 RCTs. A significant improvement in LPFS was observed in patients that had undergone breast surgery (HR = 0.37, 95% CI, 0.19-0.74; Fig. 3A). Heterogeneity between the trials was substantial (*I*^*2*^ = 83%). Moreover, 3 RCTs reported the outcome of PFS/distant PFS. Breast surgery was not associated with improved outcomes (HR = 1.14, 95% CI, 0.65-1.99; Fig. 3B). Heterogeneity between the trials was observed (*I*^*2*^ = 91%).

### Quality of Life

Two trials, ECOG EA2108^12^ and ABCSG-28,^22^ reported quality of life outcomes, while a third one, MF07-01,^23^ reported only on a subset of patients surviving at least 3 years following randomization. None of the studies reported improved quality of life outcomes with excision of the primary tumor. However, as each study employed different questionnaires to assess quality of life, meta-analysis of their results was not possible.

## Discussion

Whether removal of the breast tumor should be considered for patients with de novo MBC remains a controversial issue. With this meta-analysis including 5 RCTs and 1381 patients, we could not conclude that surgery improves OS in the total population, or in subgroups defined by receptor status or patterns of metastasis. A potential exception are younger patients, although the lack of uniform definitions and inconsistent trial results suggest that this subgroup analysis should be viewed as exploratory and requiring further validation. Expectedly, breast surgery was associated with improved locoregional disease control.

Two potential arguments in favor of surgery despite the lack of overall survival benefit are the integration of breast surgery in an effort to render oligometastatic patients clinically disease-free, and to prevent quality of life deterioration caused by local tumor overgrowth causing pain, ulceration, bleeding, and infections. Regarding oligometastatic disease, the results of the randomized phase II NRG-BR002 trial have cast doubts over the benefit of ablating all oligometastatic sites in breast cancer.^24^ This observation indicates that the results of ongoing trials examining locally aggressive therapy for oligometastatic breast cancer should be awaited. Consequently, at this time, a therapeutic approach that combines aggressive local ablation of metastases and breast surgery cannot be considered outside of clinical trials. In contrast, an exploratory analysis from PRIM-BC suggests an exacerbation of metastatic progression within 3 months following breast surgery,^13^ further highlighting our lack of understanding of the metastatic cascade and the need for carefully designed clinical trials. Regarding quality of life, none of the trials that evaluated patient-reported outcomes demonstrated any benefit in terms of patient-reported quality of life despite the significant improvement in locoregional control, providing even less grounds for routine use. Potential reasons are that the symptom burden caused by surgery may be substantial for patients treated with palliative intention,^25^ while at the same time surgery does not alleviate the psychosocial burden associated with metastatic cancer.^26^

Our meta-analysis provides the best to date available evidence regarding the role of breast surgery for patients with de novo MBC, by providing adequate power to assess all clinically relevant subgroups. Weaknesses of individual studies need, however, to be considered, as they limit the interpretation of their results. For example, imbalances in patient characteristics in the MF07-01 trial such as rates of triple negative disease and absence of histologic confirmation of solitary bone lesions favored the surgery arm,^10^ which may have confounded the reported results. In addition, protocol violations regarding planned and administered treatment have been observed, for example, in the EA2108 trial where they exceeded 10% of all patients,^12^ while at the same time no per-protocol analyses have been reported. In addition, no information on association of surgical margins with outcome was reported from most RCTs, an issue of interest due to an exploratory subgroup analysis from PRIM-BC which reported that patients operated with free margins had improved OS.^13^ Finally, systemic therapy options have advanced considerably, as the time these studies were conducted, clearly reflected in the median OS reported by the first (approximately 20 months^9^) and last published randomized trial (approximately 70 months^13^). Limitations of individual studies notwithstanding, this meta-analysis clearly demonstrates that available evidence does not support the continuous use of surgical removal of the primary tumor in patients with de novo MBC, besides the need to palliate symptoms caused by local tumor growth.

## Conclusion

By pooling data from all published RCTs, no benefit in terms of OS associated with breast surgery for patients with MBC could be demonstrated. Further trials examining this issue are ongoing (Clinicaltrials.gov identifier NCT05285332), but others have been challenged by poor accrual and early termination (NCT01392586). At this time, however, removal of the breast tumor besides the need to palliate local symptoms cannot be recommended for any subgroup of patients with the potential exception of highly selected premenopausal ones and should mainly be offered in the context of well-designed clinical trials.

## Supplementary Material

oyad266_suppl_Supplementary_Material

## Data Availability

The datasets that support the findings of this study are available from the corresponding author (A.M.) upon reasonable request.

## References

[1] Boman C , Edman Kessler L, Bergh J, Matikas A, Foukakis T. Women with short survival after diagnosis of metastatic breast cancer: a population-based registry study. Breast Cancer Res Treat. 2022;194(1):49–56. 10.1007/s10549-022-06591-7 35461374 PMC9167164

[2] Siegel RL , Miller KD, Fuchs HE, Jemal A. Cancer statistics, 2022. CA Cancer J Clin. 2022;72(1):7–33. 10.3322/caac.21708 35020204

[3] Daily K , Douglas E, Romitti PA, Thomas A. Epidemiology of de novo metastatic breast cancer. Clin Breast Cancer. 2021;21(4):302–308. 10.1016/j.clbc.2021.01.017 33750642

[4] Lord SJ , Bahlmann K, O’Connell DL, et al. De novo and recurrent metastatic breast cancer - a systematic review of population-level changes in survival since 1995. EClinicalMedicine. 2022;44:101282. 10.1016/j.eclinm.2022.101282 35128368 PMC8804182

[5] den Brok WD , Speers CH, Gondara L, et al. Survival with metastatic breast cancer based on initial presentation, de novo versus relapsed. Breast Cancer Res Treat. 2017;161(3):549–556. 10.1007/s10549-016-4080-9 28000014

[6] Karnoub AE , Dash AB, Vo AP, et al. Mesenchymal stem cells within tumour stroma promote breast cancer metastasis. Nature. 2007;449(7162):557–563. 10.1038/nature06188 17914389

[7] Petrelli F , Barni S. Surgery of primary tumors in stage IV breast cancer: an updated meta-analysis of published studies with meta-regression. Med Oncol. 2012;29(5):3282–3290. 10.1007/s12032-012-0310-0 22843291

[8] Khan SA. Primary tumor resection in stage IV breast cancer: consistent benefit, or consistent bias?. Ann Surg Oncol. 2007;14(12):3285–3287. 10.1245/s10434-007-9547-9 17891444 PMC2077920

[9] Badwe R , Hawaldar R, Nair N, et al. Locoregional treatment versus no treatment of the primary tumour in metastatic breast cancer: an open-label randomised controlled trial. Lancet Oncol. 2015;16(13):1380–1388. 10.1016/S1470-2045(15)00135-7 26363985

[10] Soran A , Ozmen V, Ozbas S, et al. Randomized trial comparing resection of primary tumor with no surgery in stage IV breast cancer at presentation: protocol MF07-01. Ann Surg Oncol. 2018;25(11):3141–3149. 10.1245/s10434-018-6494-6 29777404

[11] Fitzal F , Bjelic-Radisic V, Knauer M, et al. Impact of breast surgery in primary metastasized breast cancer: outcomes of the prospective randomized phase III ABCSG-28 POSYTIVE trial. Ann Surg. 2019;269(6):1163–1169. 10.1097/SLA.0000000000002771 31082916

[12] Khan SA , Zhao F, Goldstein LJ, et al. Early local therapy for the primary site in de novo stage IV breast cancer: results of a randomized clinical trial (EA2108). J Clin Oncol. 2022;40(9):978–987. 10.1200/JCO.21.02006 34995128 PMC8937009

[13] Shien T , Hara F, Aogi K et al. A randomized controlled trial comparing primary tumor resection plus systemic therapy with systemic therapy alone in metastatic breast cancer (PRIM-BC): Japan Clinical Oncology Group study JCOG1017. American Society of Clinical Oncology Annual Meeting 2-6 June 2023, Chicago, IL, USA. 10.1093/jjco/hys120 22833684

[14] Gennari A , Andre F, Barrios CH, et al. ESMO Clinical Practice Guideline for the diagnosis, staging and treatment of patients with metastatic breast cancer. Ann Oncol. 2021;32(12):1475–1495. 10.1016/j.annonc.2021.09.019 34678411

[15] Sabel M , Truong P. The role of local therapies in metastatic breast cancer. In Post T (ed) UpToDate. Waltham, MA Accessed on November 1 2022.

[16] Hotton J , Lusque A, Leufflen L, et al. Early locoregional breast surgery and survival in de novo metastatic breast cancer in the Multicenter National ESME Cohort. Ann Surg. 2021;277(1):e153–e161. 10.1097/sla.0000000000004767 33534229

[17] Page MJ , McKenzie JE, Bossuyt PM, et al. The PRISMA 2020 statement: an updated guideline for reporting systematic reviews. BMJ 2021;372:n71. 10.1136/bmj.n71 33782057 PMC8005924

[18] Clark JM , Sanders S, Carter M, et al. Improving the translation of search strategies using the Polyglot Search Translator: a randomized controlled trial. J Med Libr Assoc. 2020;108(2):195–207. 10.5195/jmla.2020.834 32256231 PMC7069833

[19] Bramer WM , Giustini D, de Jonge GB, Holland L, Bekhuis T. De-duplication of database search results for systematic reviews in EndNote. J Med Libr Assoc. 2016;104(3):240–243. 10.3163/1536-5050.104.3.014 27366130 PMC4915647

[20] Sterne JAC , Savovic J, Page MJ, et al. RoB 2: a revised tool for assessing risk of bias in randomised trials. BMJ. 2019;366:l4898. 10.1136/bmj.l4898 31462531

[21] Soran A , Ozmen V, Ozbas S, et al. Primary surgery with systemic therapy in patients with de novo stage IV breast cancer: 10-year follow-up; protocol MF07-01 randomized clinical trial. J Am Coll Surg. 2021;233(6):742–751.e5. 10.1016/j.jamcollsurg.2021.08.686 34530124

[22] Bjelic-Radisic V , Fitzal F, Knauer M, et al. Primary surgery versus no surgery in synchronous metastatic breast cancer: patient-reported quality-of-life outcomes of the prospective randomized multicenter ABCSG-28 Posytive trial. BMC Cancer. 2020;20(1):392. 10.1186/s12885-020-06894-2 32375735 PMC7204290

[23] Soran A , Soyder A, Ozbas S, et al. The role of loco-regional treatment in long-term quality of life in de novo stage IV breast cancer patients: protocol MF07-01Q. Support Care Cancer. 2021;29(7):3823–3830. 10.1007/s00520-020-05905-z 33242163

[24] Chmura SJ , Winter KA, Woodward WA, et al. NRG-BR002: a phase IIR/III trial of standard of care systemic therapy with or without stereotactic body radiotherapy (SBRT) and/or surgical resection (SR) for newly oligometastatic breast cancer (NCT02364557). American Society of Clinical Oncology Annual Meeting 3-7 June 2022, Chicago, IL, USA.

[25] Chow R , Pulenzas N, Zhang L, et al. Quality of life and symptom burden in patients with breast cancer treated with mastectomy and lumpectomy. Support Care Cancer. 2016;24(5):2191–2199. 10.1007/s00520-015-3027-8 26563182

[26] Girgis A , Lambert S, Johnson C, Waller A, Currow D. Physical, psychosocial, relationship, and economic burden of caring for people with cancer: a review. J Oncol Pract. 2013;9(4):197–202. 10.1200/JOP.2012.000690 23942921 PMC3710169

