# Supplementary material for: Impact of Primary Breast Surgery on Overall Survival of Patients With De Novo Metastatic Breast Cancer: A Systematic Review and Meta-Analysis
Source: Oncologist. 2023 Sep 12;29(1):1–7. doi: 10.1093/oncolo/oyad266 (PMC10769792; doi:10.1093/oncolo/oyad266)
Supplement: oyad266_suppl_Supplementary_Material [file oyad266_suppl_supplementary_material.docx]

**SUPPLEMENTARY MATERIALS**

**Table of Contents**

[Documentation of search strategies 2](#_Toc137305436)

[1. Medline 4](#_Toc137305437)

[2. Embase 5](#_Toc137305438)

[3. Web of Science Core Collection 6](#_Toc137305439)

[Supplementary Figures 7](#_Toc137305440)

[Supplementary Figure 1: Risk of bias assessment of the four randomized trials published in full form based on the RoB2 tool for the overall survival endpoint 7](#_Toc137305441)

[Supplementary Figure 2: Funnel plot and Egger’s test assessing publication bias, studies investigating the association between breast surgery and overall survival 8](#_Toc137305442)

[Supplementary Figure 3: Sensitivity analysis with pooled fixed model analysis, removal of each study, one by one, and repeating the meta-analysis 9](#_Toc137305443)

[PRISMA Checklist for the systematic review 10](#_Toc137305444)

Documentation of search strategies

University Library search consultation group

Date: July 2023

Topic/research question: Breast surgery for de novo metastatic breast cancer - does it improve prognosis compared with no surgery?

Name of researcher(s): Alexios Matikas

Librarian(s): Jonas Pettersson, Anja Vikingson, Lovisa Liljegren

Databases:

1. Medline (Ovid)
2. Embase (embase.com)
3. Web of Science Core Collection (Clarivate)

Total number of hits:

- Before deduplication: 5581
- After deduplication: 3255

Reports assessed for eligibility

(n = 1)

**Identification of studies via databases and registers**

**Identification of studies via other methods**

Records removed *before screening*:

Duplicate records removed (n = 2326)

Records marked as ineligible by automation tools (n = 0)

Records removed for other reasons (n = 0)

Records identified from*:

Databases (n = 5581)

Registers (n = 0)

Records screened

(n = 3255)

Records excluded**

(n = 3227)

Reports sought for retrieval

(n = 1)

Reports sought for retrieval

(n = 28)

Reports not retrieved

(n = 0)

**Screening**

Reports excluded:

Reports assessed for eligibility

(n = 28)

Reports excluded:

Meta-analysis (n = 4)

Retrospective study (n = 12)

Non-randomized study (n = 1)

Narrative review (n = 7)

Records identified from:

Websites (n = 328)

Organisations (n = 0)

Citation searching (n = 0)

etc.

**Identification**

Reports not retrieved

(n = 327)

Studies included in review

(n = 5)

Reports of included studies

(n = 5)

**Included**

*Consider, if feasible to do so, reporting the number of records identified from each database or register searched (rather than the total number across all databases/registers).

**If automation tools were used, indicate how many records were excluded by a human and how many were excluded by automation tools.

*From:*  Page MJ, McKenzie JE, Bossuyt PM, Boutron I, Hoffmann TC, Mulrow CD, et al. The PRISMA 2020 statement: an updated guideline for reporting systematic reviews. BMJ 2021;372:n71. doi: 10.1136/bmj.n71. For more information, visit: <http://www.prisma-statement.org/>

1. Medline

| Interface: Ovid MEDLINE(R) ALL  Date of Search: July 11 2023  Number of hits: 1114  Comment: In Ovid, two or more words are automatically searched as phrases; i.e. no quotation marks are needed | Field labels   - exp/ = exploded MeSH term - / = non exploded MeSH term - .ti,ab,kf. = title, abstract and author keywords - adjx = within x words, regardless of order - * = truncation of word for alternate endings |
| --- | --- |
| Ovid MEDLINE(R) ALL <1946 to May 16, 2023>   \| 1 \| exp Breast Neoplasms/ \| 342266 \| \| --- \| --- \| --- \| \| 2 \| exp Neoplasm Metastasis/ \| 221579 \| \| 3 \| exp Neoplasm Staging/ \| 192917 \| \| 4 \| ((breast* adj3 cancer*) or (breast* adj3 neoplasm*) or (breast* adj3 tumor*) or (breast* adj3 tumour*) or (breast* adj3 carcinoma*) or (mammar* adj3 cancer*) or (mammar* adj3 neoplasm*) or (mammar* adj3 carcinoma*)).ti,ab,kf. \| 351935 \| \| 5 \| 1 or 2 or 3 or 4 \| 735048 \| \| 6 \| exp Mastectomy/ \| 37064 \| \| 7 \| ((breast* and surger*) or mastectom* or segmentectom* or lumpectom* or quadrantectom*).ti,ab,kf. \| 60928 \| \| 8 \| 6 or 7 \| 72463 \| \| 9 \| (de novo or treatment-naive or treatment naive or first presentation or new* diagnos*).ti,ab,kf. \| 179495 \| \| 10 \| 5 and 8 and 9 \| 1114 \| | |

2. Embase

| Interface: embase.com  Date of Search: July 11 2023  Number of hits: 3031  Comment: Emtree is the controlled vocabulary in Embase | Field labels   - /exp = exploded Emtree term - /de = non exploded Emtree term - ti,ab,kw = title, abstract and author keywords - NEAR/x = within x words, regardless of order - * = truncation of word for alternate endings |
| --- | --- |
| \| No. \| Query \| Results \| \| --- \| --- \| --- \| \| #10 \| #5 AND #8 AND #9 \| 3031 \| \| #9 \| 'de novo':ti,ab,kw OR 'treatment-naive':ti,ab,kw OR 'treatment naive':ti,ab,kw OR 'first presentation':ti,ab,kw OR 'new* diagnos*':ti,ab,kw \| 331571 \| \| #8 \| #6 OR #7 \| 139064 \| \| #7 \| breast*:ti,ab,kw AND surger*:ti,ab,kw OR mastectom*:ti,ab,kw OR segmentectom*:ti,ab,kw OR lumpectom*:ti,ab,kw OR quadrantectom*:ti,ab,kw \| 113354 \| \| #6 \| 'mastectomy'/exp \| 75442 \| \| #5 \| #1 OR #2 OR #3 OR #4 \| 1692314 \| \| #4 \| ((breast* NEAR/3 cancer*):ti,ab,kw) OR ((breast* NEAR/3 neoplasm*):ti,ab,kw) OR ((breast* NEAR/3 tumor*):ti,ab,kw) OR ((breast* NEAR/3 tumour*):ti,ab,kw) OR ((breast* NEAR/3 carcinoma*):ti,ab,kw) OR ((mammar* NEAR/3 cancer*):ti,ab,kw) OR ((mammar* NEAR/3 neoplasm*):ti,ab,kw) OR ((mammar* NEAR/3 carcinoma*):ti,ab,kw) \| 585750 \| \| #3 \| 'cancer staging'/exp \| 447556 \| \| #2 \| 'metastasis'/exp \| 816936 \| \| #1 \| 'breast tumor'/exp \| 672851 \| | |

3. Web of Science Core Collection

| Interface: Clarivate Analytics  Editions = A&HCI , ESCI , SCI-EXPANDED , SSCI  Date of Search: July 11 2023  Number of hits: 1436 | Field labels   - TS/Topic = title, abstract, author keywords and Keywords Plus - NEAR/x = within x words, regardless of order - * = truncation of word for alternate endings   Note: the *Exact search*-function was used for all the searches |
| --- | --- |
| \| # \| Search Query \| Results \| \| --- \| --- \| --- \| \| 1 \| TS=(((breast* NEAR/3 cancer*) OR (breast* NEAR/3 neoplasm*) OR (breast* NEAR/3 tumor*) OR (breast* NEAR/3 tumour*) OR (breast* NEAR/3 carcinoma*) OR (mammar* NEAR/3 cancer*) OR (mammar* NEAR/3 neoplasm*) OR (mammar* NEAR/3 carcinoma*))) \| 643121 \| \| 2 \| TS=(((breast* AND surger*) OR mastectom* OR segmentectom* OR lumpectom* OR quadrantectom*)) \| 83502 \| \| 3 \| TS=(("de novo" OR treatment-naive OR "treatment naive" OR "first presentation" OR "new* diagnos*")) \| 223084 \| \| 4 \| #3 AND #2 AND #1 \| 1413 \| | |

# **Supplementary Figures**

**Supplementary Figure 1**: Risk of bias assessment of the four randomized trials published in full form based on the RoB2 tool for the overall survival endpoint


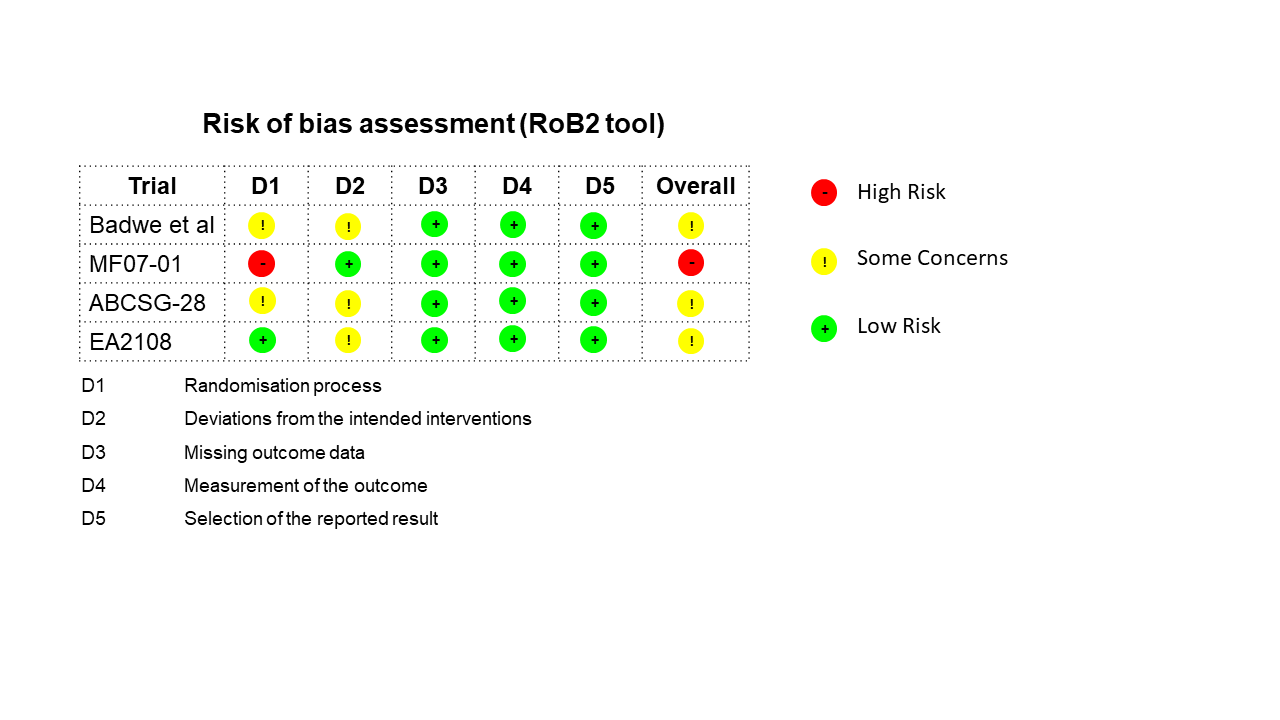


**Supplementary Figure 2**: Funnel plot and Egger’s test assessing publication bias, studies investigating the association between breast surgery and overall survival


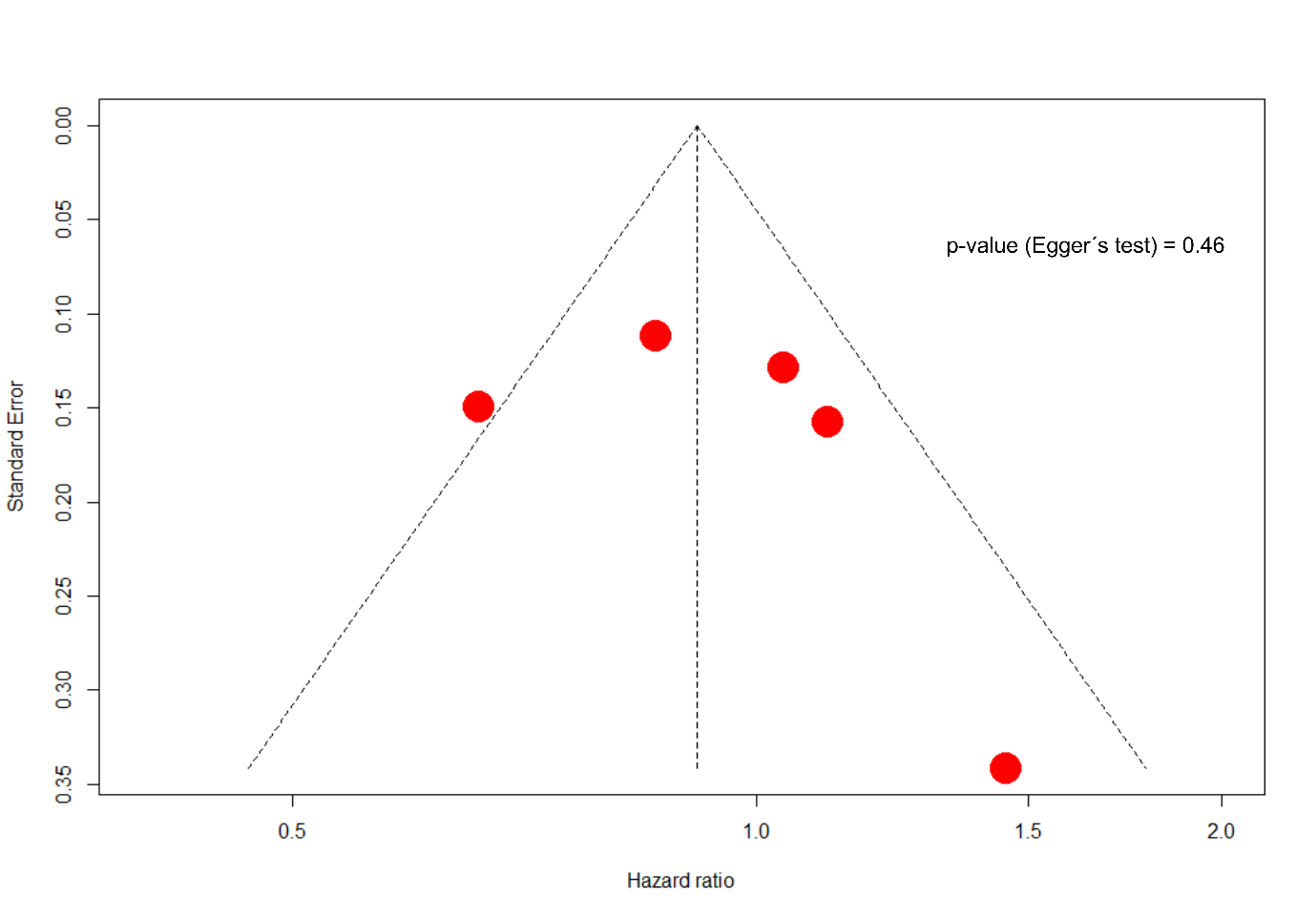


**Supplementary Figure 3**: Sensitivity analysis with pooled fixed model analysis, removal of each study, one by one, and repeating the meta-analysis

**
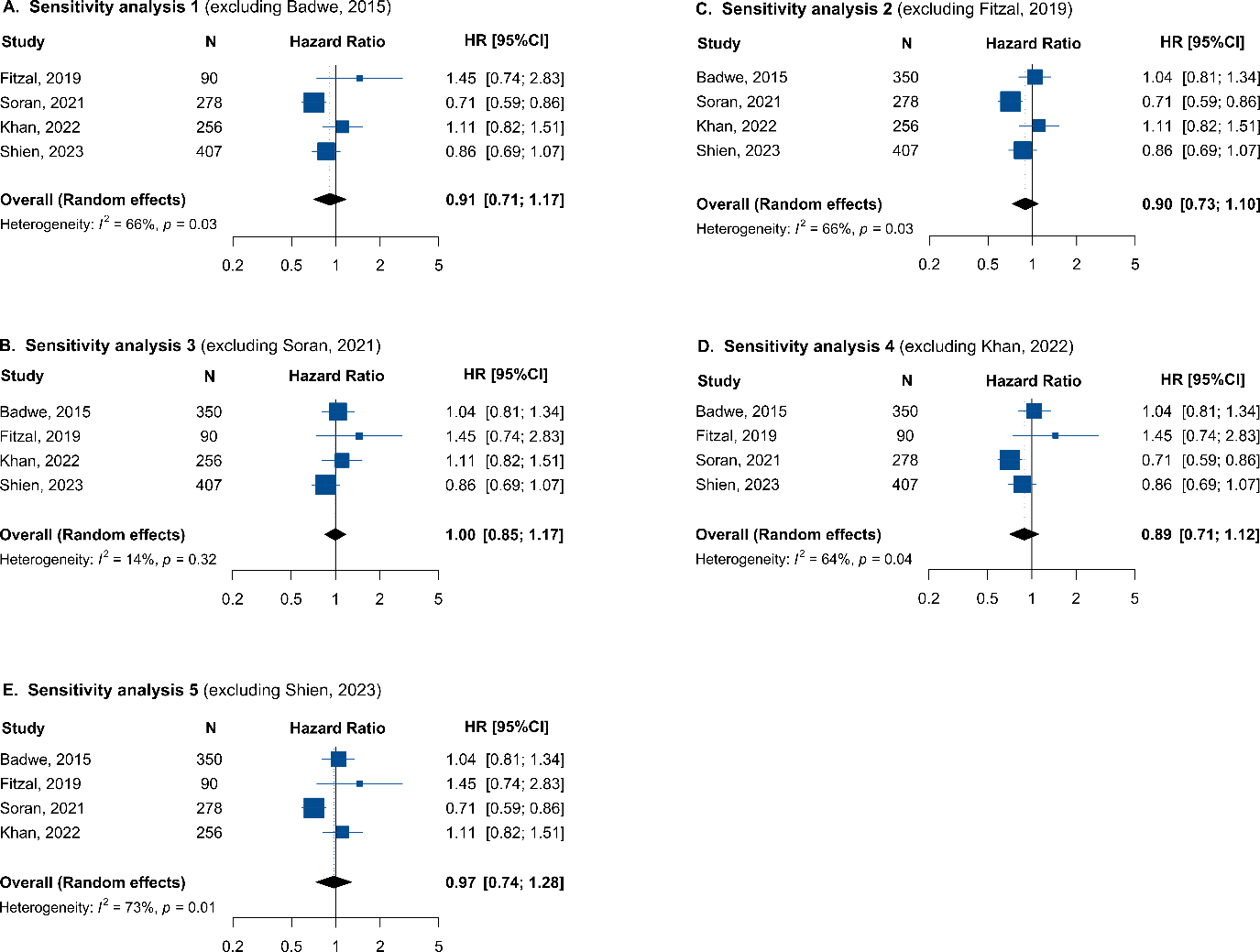
**

**PRISMA Checklist for the systematic review**

| **Section and Topic** | **Item #** | **Checklist item** | **Location where item is reported** |
| --- | --- | --- | --- |
| **TITLE** | | |  |
| Title | 1 | Identify the report as a systematic review. | Page 1 |
| **ABSTRACT** | | |  |
| Abstract | 2 | See the PRISMA 2020 for Abstracts checklist. | 2 |
| **INTRODUCTION** | | |  |
| Rationale | 3 | Describe the rationale for the review in the context of existing knowledge. | 5 |
| Objectives | 4 | Provide an explicit statement of the objective(s) or question(s) the review addresses. | 6 |
| **METHODS** | | |  |
| Eligibility criteria | 5 | Specify the inclusion and exclusion criteria for the review and how studies were grouped for the syntheses. | 7 |
| Information sources | 6 | Specify all databases, registers, websites, organisations, reference lists and other sources searched or consulted to identify studies. Specify the date when each source was last searched or consulted. | 7 |
| Search strategy | 7 | Present the full search strategies for all databases, registers and websites, including any filters and limits used. | Supplement |
| Selection process | 8 | Specify the methods used to decide whether a study met the inclusion criteria of the review, including how many reviewers screened each record and each report retrieved, whether they worked independently, and if applicable, details of automation tools used in the process. | 8 |
| Data collection process | 9 | Specify the methods used to collect data from reports, including how many reviewers collected data from each report, whether they worked independently, any processes for obtaining or confirming data from study investigators, and if applicable, details of automation tools used in the process. | 8 |
| Data items | 10a | List and define all outcomes for which data were sought. Specify whether all results that were compatible with each outcome domain in each study were sought (e.g. for all measures, time points, analyses), and if not, the methods used to decide which results to collect. | 9 |
|  | 10b | List and define all other variables for which data were sought (e.g. participant and intervention characteristics, funding sources). Describe any assumptions made about any missing or unclear information. | 8 |
| Study risk of bias assessment | 11 | Specify the methods used to assess risk of bias in the included studies, including details of the tool(s) used, how many reviewers assessed each study and whether they worked independently, and if applicable, details of automation tools used in the process. | 8 |
| Effect measures | 12 | Specify for each outcome the effect measure(s) (e.g. risk ratio, mean difference) used in the synthesis or presentation of results. | 9 |
| Synthesis methods | 13a | Describe the processes used to decide which studies were eligible for each synthesis (e.g. tabulating the study intervention characteristics and comparing against the planned groups for each synthesis (item #5)). | 7 |
|  | 13b | Describe any methods required to prepare the data for presentation or synthesis, such as handling of missing summary statistics, or data conversions. | N/A |
|  | 13c | Describe any methods used to tabulate or visually display results of individual studies and syntheses. | 9 |
|  | 13d | Describe any methods used to synthesize results and provide a rationale for the choice(s). If meta-analysis was performed, describe the model(s), method(s) to identify the presence and extent of statistical heterogeneity, and software package(s) used. | 9 |
|  | 13e | Describe any methods used to explore possible causes of heterogeneity among study results (e.g. subgroup analysis, meta-regression). | 10 |
|  | 13f | Describe any sensitivity analyses conducted to assess robustness of the synthesized results. | 9-10 |
| Reporting bias assessment | 14 | Describe any methods used to assess risk of bias due to missing results in a synthesis (arising from reporting biases). | 9-10 |
| Certainty assessment | 15 | Describe any methods used to assess certainty (or confidence) in the body of evidence for an outcome. | N/A |
| **RESULTS** | | |  |
| Study selection | 16a | Describe the results of the search and selection process, from the number of records identified in the search to the number of studies included in the review, ideally using a flow diagram. | 11, supplement |
|  | 16b | Cite studies that might appear to meet the inclusion criteria, but which were excluded, and explain why they were excluded. | N/A |
| Study characteristics | 17 | Cite each included study and present its characteristics. | Table 1 |
| Risk of bias in studies | 18 | Present assessments of risk of bias for each included study. | Supplement |
| Results of individual studies | 19 | For all outcomes, present, for each study: (a) summary statistics for each group (where appropriate) and (b) an effect estimate and its precision (e.g. confidence/credible interval), ideally using structured tables or plots. | Figures 1-3 |
| Results of syntheses | 20a | For each synthesis, briefly summarise the characteristics and risk of bias among contributing studies. | Supplement |
|  | 20b | Present results of all statistical syntheses conducted. If meta-analysis was done, present for each the summary estimate and its precision (e.g. confidence/credible interval) and measures of statistical heterogeneity. If comparing groups, describe the direction of the effect. | Figures 1-3 |
|  | 20c | Present results of all investigations of possible causes of heterogeneity among study results. | Figures 1-3 |
|  | 20d | Present results of all sensitivity analyses conducted to assess the robustness of the synthesized results. | 12 |
| Reporting biases | 21 | Present assessments of risk of bias due to missing results (arising from reporting biases) for each synthesis assessed. | N/A |
| Certainty of evidence | 22 | Present assessments of certainty (or confidence) in the body of evidence for each outcome assessed. | N/A |
| **DISCUSSION** | | |  |
| Discussion | 23a | Provide a general interpretation of the results in the context of other evidence. | 14 |
|  | 23b | Discuss any limitations of the evidence included in the review. | 15 |
|  | 23c | Discuss any limitations of the review processes used. | N/A |
|  | 23d | Discuss implications of the results for practice, policy, and future research. | 14-16 |
| **OTHER INFORMATION** | | |  |
| Registration and protocol | 24a | Provide registration information for the review, including register name and registration number, or state that the review was not registered. | 7 |
|  | 24b | Indicate where the review protocol can be accessed, or state that a protocol was not prepared. | N/A |
|  | 24c | Describe and explain any amendments to information provided at registration or in the protocol. | N/A |
| Support | 25 | Describe sources of financial or non-financial support for the review, and the role of the funders or sponsors in the review. | 21 |
| Competing interests | 26 | Declare any competing interests of review authors. | 21 |
| Availability of data, code and other materials | 27 | Report which of the following are publicly available and where they can be found: template data collection forms; data extracted from included studies; data used for all analyses; analytic code; any other materials used in the review. | 21 |

**Note: Page numbers correspond to the original submission**
